# Recent Discoveries in Nanoparticle–Macrophage Interactions: In Vitro Models for Nanosafety Testing and Novel Nanomedical Approaches for Immunotherapy

**DOI:** 10.3390/nano11112971

**Published:** 2021-11-05

**Authors:** Fernando Torres Andón, Olesja Bondarenko

**Affiliations:** 1 Center for Research in Molecular Medicine & Chronic Diseases (CiMUS), Universidade de Santiago de Compostela, Campus Vida, 15706 Santiago de Compostela, Spain; 2 IRCCS Istituto Clinico Humanitas, Via A. Manzoni 56, Rozzano, 20089 Milan, Italy; 3 National Institute of Chemical Physics and Biophysics, Akadeemia tee 23, 12618 Tallinn, Estonia; 4 Institute of Biotechnology, HiLIFE, University of Helsinki, Viikinkaari 5d, 00790 Helsinki, Finland

Nanoparticles (NPs) offer unique properties for biomedical applications, leading to new nanomedicines. Recent examples of advanced nanoparticle-based nanomedicines are COVID-19 RNA vaccines. Regardless of the delivery route of the NPs into the body (intravenous or subcutaneous injection, oral, intranasal, etc.), NPs inevitably come into contact with immune cells, such as macrophages. Macrophages are phagocytizing cells that determine the fate and the lifetime of NPs in relevant biological fluids or tissues, which has consequences for both nanosafety and nanomedicine.

The aim of this Special Issue is to cover recent advancements in our understanding of NP–macrophage interactions, with a focus on in vitro models for nanosafety and novel nanomedicine approaches that allow the modulation of the immunological profile of macrophages. The current Special Issue compiles nine papers: seven research articles and two review articles. The original articles include studies on the interaction of different nanomaterials, such as multi-walled carbon nanotubes (MWCNTs) [1,2,3], amorphous silica [4], gold nanoparticles [3,5], lipid carriers [6], and microspheres [7], with macrophages in different scenarios.

Most of the articles in this Special Issue are focused on the toxicological aspects of nanomaterials and in vitro models. Wang et al. found that carboxylated-MWCNTs (cMWCNTs) coated with Pluronic^®^F-108 (PF108) interacted with the class A scavenger receptor (SR-A1) on the surface of alveolar macrophages, whereas both pristine-MWCNTs (pMWCNTs) and amino-functionalized-MWCNTs coated with PF108 did not. This interaction was crucial for the uptake of cMWCNTs by macrophages resulting in higher toxicity and impaired phagocytic activity for other SR-A1 ligands. Similarly, Huynh et al. [2] coated cMWCNTs and pMWCNTs with bovine serum albumin (BSA) and observed an increased uptake for BSA-cMWCNTs versus BSA-pMWCNTs by RAW264.7 macrophages. The authors hypothesized that SR-A1 can interact with two structural features of BSA-cMWNTs, one inherent to the oxidized nanotubes and the other provided by the BSA corona. In another work, Wiemann et al. studied the interaction of serum-coated synthetic amorphous silica (SAS) NPs with alveolar NR8383 macrophages. While under serum-free conditions, SAS NPs were taken up by macrophages, which resulted in toxicity; these effects were mitigated by the presence of serum [4]. Similar results were previously observed by Gallud et al. [8] with mesoporous silica, where the cytotoxicity of the NPs was mitigated in the presence of serum. These toxicological studies have implications for the nanosafety of biomaterials, but also for the potential application of NPs in medicine.

Depending on the route of administration [9], NPs interact with different proteins and form a protein corona that heavily influences their interaction with macrophages [10]. Thus, studies on the interaction of NPs with proteins and macrophages with different phenotypes are of foremost importance. Upon the interaction and uptake of NPs by macrophages, different outcomes are possible [11]. In this Special Issue, Keshavan et al. [3] published an in vitro study comparing three benchmark nanomaterials (Ag NPs, TiO_2_ NPs, and MWCNTs) procured from the nanomaterial repository at the Joint Research Centre of the European Commission. The exposure of sub-lethal concentrations of macrophage-like cell line (THP-1 pretreated with phorbol-12-myristate-13-acetate) to these NPs induced changes in mRNA expression that was mostly related to immune networks such as cytokine and chemokine signaling pathways. Using sub-lethal concentrations of MWCNTs, modest inductions of IL-6 and IL-1β were observed, while CCR2-CCL2 was identified as the most significantly upregulated pathway [3]. This activation of CCL2, also referred to as monocyte chemoattractant protein-1 (MCP-1), may have a role in the granuloma formation in the lungs, pleural or abdominal cavity following exposure to MWCNTs. In previous studies, we also reported on the secretion of IL-1β by hollow carbon spheres [12], or the specific interaction of single-walled carbon nanotubes (SWCNTs) with TLR2 in the absence of protein corona [13]. Of foremost importance, we demonstrate that these types of experiments must be combined with biodegradation studies in order to understand the benefits and limitations of new nanotechnologies, such as carbon nanotubes [14], for their safe application for industrial or medical purposes.

For immunotoxicology studies, in silico and in vitro methods, including cell- and organ-based assays, are encouraged. Mimicking the immune system in vitro is not easy, and important differences have been observed between two- (2D) and three-dimensional (3D) cell cultures. In this Special Issue, Swartzwelter et al. performed a comprehensive comparison of traditional 2D cultures with 3D collagen-matrix models, representing the skin, including human blood monocytes exposed to gold nanoparticles (AuNPs) [5]. Immediate inflammation related to the stimulation of fresh monocytes, and the secondary reaction of monocyte-derived macrophages after previous priming, were evaluated. They observed similar TNF-α and IL-6 responses in 2D and 3D cultures, but notable differences in IL-8 or IL-1Rα, mainly in the recall/memory response of primed cells to a second stimulation, with the 3D cultures showing a clearer cell activation and memory effect in response to AuNPs. Although our knowledge of innate memory is still largely incomplete, it has been demonstrated that its activation is involved in defensive responses or can lead to a pathological exacerbation of secondary reactions. Furthermore, the therapeutic manipulation of this innate memory using different drugs or even NPs was postulated for medical purposes [15]. Thus, the inclusion of macrophages and the allowing of their long-term culture in vitro in these types of 3D models is crucial for the improved testing of new NPs.

As a step forward in the screening of NPs, but still avoiding the use of mammalians, zebrafish models present important ethical and economic advantages. In the review conducted by Pensado-López et al., we learn that zebrafish (Danio rerio) now constitute a well-established model for the toxicological and pharmacological screening of new drugs and nanomaterials, thanks to their rapid embryo development, small size and transparency, and genetic and physiological conservation [16]. Zebrafish with fluorescently labeled macrophages, including disease models that cause and/or are caused by inflammatory disorders (i.e., cancer, autoimmune or infectious diseases) have been developed. The number of publications tracking the interaction between macrophages and NPs using zebrafish has been clearly increasing in recent years, and it is expected that this research will lead to further knowledge about the role of macrophages in the initiation, progression, and remission of diseases over the course of a treatment, but will also contribute to the safe use of NPs and their translation towards the clinic.

In this Special Issue, we also included some manuscripts with a focus on the therapeutic application of nanotechnological approaches. We presented a review on the most recent nano-based drug delivery strategies to manipulate the immune system in the context of osteoarthritis, with a particular focus on those designed to specifically target and reprogram macrophages [17]. In the context of the joint pathology, macrophages with an M1-like pro-inflammatory phenotype induce chronic inflammation and joint destruction, and they have been correlated with the development and progression of the disease, while the M2-like anti-inflammatory macrophages support the recovery of the disease, promoting tissue repair and the resolution of inflammation. Thus, the use of NPs, liposomes, or hybrid nanosystems to locally improve the delivery of anti-inflammatory drugs to macrophages has been investigated. An interesting approach for wound healing was reported by Santos-Vizcaino et al. [7]. They studied the mechanism of action of negatively charged microspheres (NCM) from a commercial formulation to revert the chronic inflammatory state of stagnant wounds, such as diabetic wounds. After toxicity experiments, they demonstrated the internalization of NCMs by macrophages, driving their polarization towards an anti-inflammatory M2-phenotype that favors the wound-healing processes.

With a different therapeutic purpose, Rouco et al. evaluated, in vitro, the activity of rifabutin-loaded lipid-nanocarriers (RFB-NLC) [6]. These RFB-NLCs showed macrophage uptake and selective intracellular release of RFB, thus constituting a promising strategy to improve oral anti-mycobacterial therapy in Crohn’s disease. Although in vivo experiments were not performed, the authors hypothesize that the passage and accumulation of NPs in the intestinal inflamed sites, densely infiltrated by macrophages, might be favored by the disruption of the epithelial barrier observed in inflammatory bowel disease patients.

Overall, as Editors of Nanomaterials, we are fully aware that the present Special Issue cannot fully reflect the high number and diversity of studies on nanoparticle–macrophage interactions. Thus, we also encourage the reading of other general reviews on nanotoxicology and nanomedicine [18], and specific reviews on the interaction of NPs with macrophages for the treatment of cancer [10], infectious diseases [19], other inflammatory disorders [15,20] such as the COVID-19-related cytokine storm [21], or strategies to improve the biocompatibility of antibacterial NPs [22].

In summary, we are confident that this Special Issue will contribute to the research interest in the field, providing our readership with a multi-faceted scenario that outlines the importance of macrophage-based in vitro models for nanosafety, and awareness about novel therapeutic approaches, such as the reprogramming of macrophages, using nanomedicines.
